# Pre-frailty after blood or marrow transplantation and the risk of subsequent mortality

**DOI:** 10.1038/s41375-024-02238-2

**Published:** 2024-04-05

**Authors:** Nora Balas, Joshua S. Richman, Wendy Landier, Sadeep Shrestha, Katia J. Bruxvoort, Lindsey Hageman, Qingrui Meng, Elizabeth Ross, Alysia Bosworth, F. Lennie Wong, Ravi Bhatia, Stephen J. Forman, Saro H. Armenian, Daniel J. Weisdorf, Smita Bhatia

**Affiliations:** 1 University of Alabama at Birmingham, Birmingham, AL USA; 2 City of Hope, Duarte, CA USA; 3 University of Minnesota, Minneapolis, MN USA

**Keywords:** Epidemiology, Signs and symptoms

## Abstract

We examined the prevalence, risk factors, and association between pre-frailty and subsequent mortality after blood or marrow transplantation (BMT). Study participants were drawn from the BMT Survivor Study (BMTSS) and included 3346 individuals who underwent BMT between 1974 and 2014 at one of three transplant centers and survived ≥2 years post-BMT. Participants completed the BMTSS survey at a median of 9 years from BMT and were followed for subsequent mortality for a median of 5 years after survey completion. Closest-age and same-sex biological siblings also completed the survey. Previously published self-reported indices (exhaustion, weakness, low energy expenditure, slowness, unintentional weight loss) classified participants as non-frail (0–1 indices) or pre-frail (2 indices). National Death Index was used to determine vital status and cause of death. Overall, 626 (18.7%) BMT survivors were pre-frail. BMT survivors had a 3.2-fold higher odds of being pre-frail (95% CI = 1.9–5.3) compared to siblings. Compared to non-frail survivors, pre-frail survivors had higher hazards of all-cause mortality (adjusted hazard ratio [aHR] = 1.6, 95% CI = 1.4–2.0). Female sex, pre-BMT radiation, smoking, lack of exercise, anxiety, and severe/life-threatening chronic health conditions were associated with pre-frailty. The novel association between pre-frailty and subsequent mortality provides evidence for interventions as pre-frail individuals may transition back to their robust state.

## Introduction

Frailty is characterized by exhaustion, weakness, low physical activity, slow walking speed, and unintentional weight loss. Frail individuals exhibit three or more of these indices, while pre-frail individuals exhibit two indices [1]. Blood or marrow transplantation (BMT) is used with curative intent for hematologic malignancies and other life-threatening illnesses. High-intensity therapeutic exposures, chronic graft *vs* host disease (cGvHD), and treatment-related morbidity serve as substantial stressors and increase the risk of frailty among BMT survivors. While the association between frailty and subsequent mortality is clearly established among BMT survivors [1], there is limited attention to the pre-frail state, although there is emerging evidence in the general population that pre-frail individuals are also at increased risk for subsequent mortality [2]. There is evidence among community-dwelling adults that pre-frail individuals are more likely to transition back to a robust state compared to those who are frail [3, 4].

Given the elevated risk of mortality among pre-frail individuals in the general population, and the higher probability of returning to the non-frail state for pre-frail rather than frail individuals, targeting the pre-frail state for intervention may be considered optimal. This underscores the importance of understanding the prevalence of pre-frailty and associated risk factors in BMT survivors, and the association of pre-frailty with subsequent mortality. The present study addresses these gaps using the resources offered by the BMT Survivor Study (BMTSS).

## Materials and methods

### Study participants and data collection

BMTSS was established to examine the long-term outcomes of individuals who survived ≥2 years after undergoing BMT between 1974 and 2014 at the University of Alabama at Birmingham (UAB), City of Hope (COH) or University of Minnesota (UMN). BMTSS also examines outcomes in an unaffected comparison group drawn from the survivors’ siblings. Study participation consists of completion of the BMTSS survey by the BMT survivors and siblings. The survey captures sociodemographic characteristics (sex, race/ethnicity, education, annual household income, and health insurance), chronic health conditions (as diagnosed by their healthcare provider), history of chronic graft vs. host disease (cGvHD), relapse and subsequent neoplasms after BMT, health risk behaviors (smoking, alcohol consumption, and lack of exercise) and BMT-related anxiety (Supplementary Table 1). Chronic health conditions have been graded using the Common Terminology Criteria for Adverse Events, v5.0 from grade 1 (mild) to grade 5 (death due chronic health condition) [5]. Survivors’ age at BMT, primary diagnosis, type of BMT (autologous, allogeneic), risk of relapse at BMT (standard risk: first or second complete remission after acute lymphoblastic [ALL] or acute myeloid leukemia [AML], Hodgkin lymphoma [HL] or non-Hodgkin lymphoma [NHL], first chronic phase of chronic myeloid leukemia [CML], or severe aplastic anemia; high risk: all other patients) [6], stem cell source (bone marrow, peripheral blood stem cells [PBSCs], or cord blood), use of total body irradiation (TBI) for conditioning, conditioning intensity (myeloablative conditioning [MAC] or non-myeloablative/reduced-intensity conditioning [collectively termed NMA]), and pre-BMT therapeutic exposures were retrieved from institutional transplant databases and medical records. BMT survivors were placed into four groups based on TBI exposure and conditioning intensity: NMA/no TBI, NMA/TBI, MAC/no TBI, and MAC/TBI. The Institutional Review Board (IRB) at UAB serves as the single IRB of record; IRBs at UMN and COH approved the BMTSS protocol. Participants provided informed consent according to the Declaration of Helsinki. The present report includes survivors who received BMT at any age and were 18 years of age or older when they completed the BMTSS survey. Siblings were also 18 years or older at study participation.

### Frailty phenotype

Frailty phenotype was constructed from responses provided by BMT survivors for the following five indices (Supplementary Table 2): clinically underweight, exhaustion, low energy expenditure, slowness, and muscle weakness. Participants were categorized as frail (≥3 indices), pre-frail (2 indices), or non-frail (0–1 indices) [1]. For this analysis, we excluded survivors and siblings who met the criteria for frailty and retained only those who were pre-frail or non-frail.

### Late mortality

The primary outcomes of interest included all-cause and cause-specific late mortality (non-recurrence-related [NRM] and recurrence-related mortality [RRM]). National Death Index (NDI) Plus provided data regarding the date and cause of death through December 31, 2020 [7]. Additional data from the Accurint database [8] extended the vital status information through December 21, 2021. Suicides, homicides, and accidents were classified as external causes of death. A cause of death matching the pre-transplant diagnosis was classified as RRM. All other causes of death were classified as NRM (subsequent malignant neoplasms [SMNs], cardiovascular disease [CVD], infections, pulmonary, etc.).

### Statistical analysis

#### Comparison with same-sex biological siblings

We paired closest-age same-sex biological siblings with BMT survivors. To estimate adjusted odds ratios (aOR) and 95% confidence intervals (95% CI) of pre-frailty in BMT survivors compared with their siblings we used logistic regression with generalized estimating equation [9] to account for the paired data. The following variables were evaluated for inclusion in the model: age at survey grades 3–4 chronic health conditions, socioeconomic status (SES: <college and <$50,000; <college and ≥$50,000; ≥college and <$50,000; ≥college and ≥$50,000), health insurance, alcohol consumption, lack of exercise and smoking.

#### Factors associated with pre-frailty among the BMT survivors

Risk factors examined for association with pre-frailty included time from BMT to survey, age at survey, sex, race/ethnicity, SES, health insurance, primary diagnosis, pre-BMT radiation, risk of relapse at BMT, BMT era (1974–1989; 1990–2004; 2005–2014) [10], BMT institution (UAB; COH; UMN), stem cell source, conditioning intensity/TBI, BMT type/cGvHD (autologous BMT; allogeneic BMT/no cGvHD; allogeneic BMT/cGvHD), grades 3–4 chronic health conditions, post-BMT relapse, BMT-related anxiety, smoking, alcohol consumption, and lack of physical activity. These demographic and clinical variables were examined for their possible inclusion in the multivariable analysis based on their significance in the unadjusted model and previous knowledge (Supplementary Table 3); associations between the risk factors and pre-frailty were reported as unadjusted OR with corresponding 95% CI, using non-frail as the reference group.

#### Pre-frailty status and subsequent mortality among BMT survivors

Kaplan–Meier methods were used to calculate overall survival. Cox proportional hazards models with time from survey as the time axis was used for identifying the association between pre-frailty and all-cause mortality. Demographic and clinical variables listed above were examined for their possible inclusion in the models (Supplementary Table 4). We examined statistical interaction between pre-frailty and key risk factors (primary disease, chronic health conditions, stem cell source, age at BMT [<45 years; ≥45 years], age at survey [<65 years; ≥65 years], and BMT type), to assess if the association between pre-frailty and subsequent mortality was modified by these variables.

Cumulative incidence of cause-specific mortality was calculated using competing risk methods; deaths attributed to RRM and external causes served as competing risks for NRM, and deaths attributed to NRM and external causes served as competing risks for RRM. Proportional sub-distribution hazard (Fine-Gray) models [11] were used to examine the association between pre-frailty and cause-specific mortality. Participants with missing or unknown cause of death were excluded from the proportional sub-distribution hazard models for RRM and NRM. Adjustment of the models with demographic and clinical factors was similar to that described in the Cox regression models above. Results were presented as adjusted hazard ratio (aHR) with 95% CI.

All analyses were performed using SAS v9.4 (SAS Institute, Cary, North Carolina). Findings with 2-sided tests were considered statistically significant at *P* < 0.05.

## Results

Of the 5765 eligible BMT survivors approached, 4253 (73.8%) consented and completed the survey. We had to exclude 387 participants who completed abbreviated version of the BMTSS survey that did not ask questions related to frailty. An additional 41 participants were excluded because they did not complete the questions related to frailty, and 479 frail participants were excluded, yielding 3346 evaluable BMT survivors (Supplementary Fig. 1). Compared with those who refused participation, participants were more likely to be non-Hispanic White (75.4% *vs*. 63.8%; *P* < *0.0001*), more likely to have received MAC/TBI (38.3% *vs*. 26.5%; *P* < *0.0001*) and were older at BMT (mean age, 42 years *vs*. 38y; *P* < *0.0001*) (Supplementary Table 5).

As shown in Table 1, 44.4% of the survivors were female, 73.7% were <65 years at survey completion, and 75.4% were non-Hispanic White. The median age at BMT was 45 years (IQR: 29–56), 49.1% had received an allogeneic BMT, and 38.3% had received MAC/TBI. The indications for BMT included HL/NHL (32.8%), AML/MDS (23.5%), plasma cell dyscrasias (PCD: 17.1%), CML (10%), ALL (8.3%), and other diagnoses (8.2%). Grades 3–4 chronic health conditions were reported by 56.4% of the survivors. Median interval between BMT and survey completion was 9 years (IQR: 5–15), and between survey completion and death/end of follow-up (December 2021) was 5 years (IQR: 4–7). Overall, 626 (18.7%) BMT survivors were pre-frail.

**Table 1:** Clinical characteristics of BMT survivors by pre-frailty status.

Variables of interest	Total *N* = 3346	Pre-frailty status	
		Non-Frail *N* = 2720 (81.3%)	Pre-Frail *N* = 626 (18.7%)
Age at completing the survey in years			
Median (IQR)	57 (44–65)	56 (43–65)	57 (45–65)
Age at completing the survey in years, *n* (%)			
<65	2467 (73.7)	2009 (73.9)	458 (73.2)
≥65	879 (26.3)	711 (26.1)	168 (26.2)
Follow-up from completing the survey to death or end of follow-up in years			
Median (IQR)	5 (4–7)	5 (4–7)	5 (4–7)
BMT era, *n* (%)			
1974–1989	283 (8.4)	234 (8.6)	49 (7.8)
1990–2004	1447 (43.3)	1177 (43.3)	270 (43.1)
2005–2014	1616 (48.3)	1309 (48.1)	307 (49.0)
Institution, *n* (%)			
COH	2057 (61.5)	1652 (60.7)	405 (64.7)
UMN	1051 (31.4)	880 (32.3)	171 (27.3)
UAB	238 (7.1)	188 (6.9)	50 (8.0)
Sex, *n* (%)			
Female	1485 (44.4)	1175 (43.2)	310 (49.5)
Male	1861 (55.6)	1545 (56.8)	316 (50.5)
Race/Ethnicity, *n* (%)			
African American	154 (4.6)	131 (4.8)	23 (3.7)
Non-Hispanic White	2522 (75.4)	2032 (74.7)	490 (78.3)
Hispanic	401 (12.0)	340 (12.5)	61 (9.7)
Asian	178 (5.3)	142 (5.2)	36 (5.7)
Other^a^	88 (2.6)	73 (2.7)	15 (2.4)
Missing	3 (0.1)	2 (0.1)	1 (0.2)
Health insurance, *n* (%)			
Not insured	123 (3.7)	104 (3.8)	19 (3.0)
Insured	3217 (96.1)	2612 (96.0)	605 (96.7)
Missing	6 (0.2)	4 (0.1)	2 (0.3)
Socioeconomic status, *n* (%)			
<College and <$50,000	497 (14.8)	399 (14.7)	98 (15.6)
<College and ≥$50,000	218 (6.5)	177 (6.5)	41 (6.5)
≥College and <$50,000	1001 (29.9)	789 (29.0)	212 (33.9)
≥College and ≥$50,000	1322 (39.5)	1110 (40.8)	212 (33.9)
Missing education and/or income	308 (9.2)	245 (9.0)	63 (10.1)
Age at BMT in years			
Median (IQR)	45 (29–56)	44 (28–56)	47 (32–56)
Follow-up from BMT to completing the survey in years			
Median (IQR)	9 (5–15)	9 (6–15)	8 (5–14)
Primary diagnosis, *n* (%)			
ALL	279 (8.3)	224 (8.2)	55 (8.8)
AML/MDS	785 (23.5)	634 (23.3)	151 (24.1)
CML	335 (10.0)	276 (10.1)	59 (9.4)
HL	268 (8.0)	223 (8.2)	45 (7.2)
NHL	830 (24.8)	688 (25.3)	142 (22.7)
PCD	573 (17.1)	442 (16.2)	131 (20.9)
Other^b^	276 (8.2)	233 (8.6)	43 (6.9)
Risk of relapse at first BMT, *n* (%)			
High risk	1466 (43.8)	1158 (42.6)	308 (49.2)
Standard risk	1487 (44.4)	1226 (45.1)	261 (41.7)
Missing	393 (11.8)	336 (12.3)	57 (9.1)
Post-BMT relapse, *n* (%)			
No	3001(89.7)	2453 (90.2)	548 (87.5)
Yes	263 (7.8)	206 (7.6)	57 (9.1)
Missing	82 (2.5)	61 (2.2)	21 (3.4)
BMT type/ cGvHD, *n* (%)			
Autologous	1704 (50.9)	1369 (50.3)	335 (53.5)
Allogeneic with cGvHD	852 (25.5)	689 (25.3)	163 (26.0)
Allogeneic without cGvHD	744 (22.2)	624 (22.9)	120 (19.2)
Allogeneic missing cGvHD	46 (1.4)	38 (1.4)	8 (1.3)
Stem cell source, *n* (%)			
Bone Marrow/Cord Blood	1135 (33.9)	952 (35.0)	183 (29.2)
Peripheral Stem Cells	2210 (66.0)	1767 (65.0)	443 (70.8)
Missing	1 (0.1)	1 (0.04)	0 (0.0)
Conditioning intensity/ Total Body Irradiation, *n* (%)			
MAC/no TBI	1048 (31.3)	863 (31.7)	185 (29.5)
MAC/TBI	1283 (38.3)	1039 (38.2)	244 (39.0)
NMA/no TBI	435 (13.0)	353 (13.0)	82 (13.1)
NMA/TBI	217 (6.5)	180 (6.6)	37 (5.9)
Missing	363 (10.9)	285 (10.5)	78 (12.5)
Pre-BMT radiation, *n* (%)			
Yes	470 (14.1)	360 (13.2)	110 (17.6)
No	2597 (77.6)	2124 (78.1)	473 (75.6)
Missing	279 (8.3)	236 (8.7)	43 (6.9)
Chronic health conditions, *n* (%)			
Grades 3 or 4	1886 (56.4)	1463 (53.8)	423 (67.6)
BMT-related anxiety, *n* (%)			
Present	114 (3.4)	72 (2.6)	42 (6.7)
Absent	3194 (95.5)	2620 (96.3)	574 (91.7)
Missing	38 (1.1)	28 (1.0)	10 (1.6)
Smoking status, *n* (%)			
Never smoker	2132 (63.7)	1770 (65.1)	362 (57.8)
Ever smoker	1197 (35.8)	937 (34.4)	260 (41.5)
Missing	17 (0.5)	13 (0.5)	4 (0.6)
Alcohol consumption status, *n* (%)			
Non-drinker	1433 (42.8)	1146 (42.1)	287 (45.9)
Non-heavy drinker	1729 (51.7)	1422 (52.3)	307 (49.0)
Heavy drinker	165 (4.9)	138 (5.1)	27 (4.3)
Missing	19 (0.6)	14 (0.5)	5 (0.8)
Lack of exercise *n* (%)			
Yes	508 (15.2)	355 (13.1)	153 (24.5)
No	2825 (84.4)	2354 (86.5)	471 (75.2)
Missing	13 (0.4)	11 (0.4)	2 (0.3)
Cause of death^c^, *n* (%)			
Non-recurrence	305 (9.1)	218 (48.2)	87 (47.3)
Recurrence	183 (5.4)	130 (28.8)	53 (28.8)
Unknown	135 (4.0)	94 (20.8)	41 (22.3)
External	13 (0.4)	10 (2.2)	3 (1.6)

*BMT* Blood or Marrow Transplantation, *AML* Acute Myeloid Leukemia, *MDS* Myelodysplastic Syndrome, *ALL* Acute lymphoblastic leukemia, *HL* Hodgkin’s Lymphoma, *NHL* Non-Hodgkin’s lymphoma, *PCD* plasma cell dyscrasias, *CML* chronic myeloid leukemia, *cGvHD* Chronic Graft vs Host Disease, *MAC* Myeloablative, *NMA* Non-Myeloablative, *TBI* Total Body irradiation, *UAB* University of Alabama at Birmingham, *COH* City of Hope (COH), *UMN* University of Minnesota, *IQR* Interquartile range.

^a^Race “other” includes Multiracial (*n* = 75), American Indian (*n* = 11), Pacific Island (*n* = 2).

^b^Primary diagnosis “other” includes severe aplastic anemia (SAA), other Leukemia.

^c^Among the deceased participants (*n* = 636), non-recurrence-related mortality includes second malignant neoplasms (*n* = 111), cardiac (*n* = 69), infection (*n* = 59), pulmonary (*n* = 19), renal (*n* = 13), stroke (*n* = 12), neurologic (*n* = 7), other (*n* = 6), hemorrhage (*n* = 5), hepatic (*n* = 3), venous thromboembolism (*n* = 1).

### Comparison with biological siblings

**Fig. 1:**
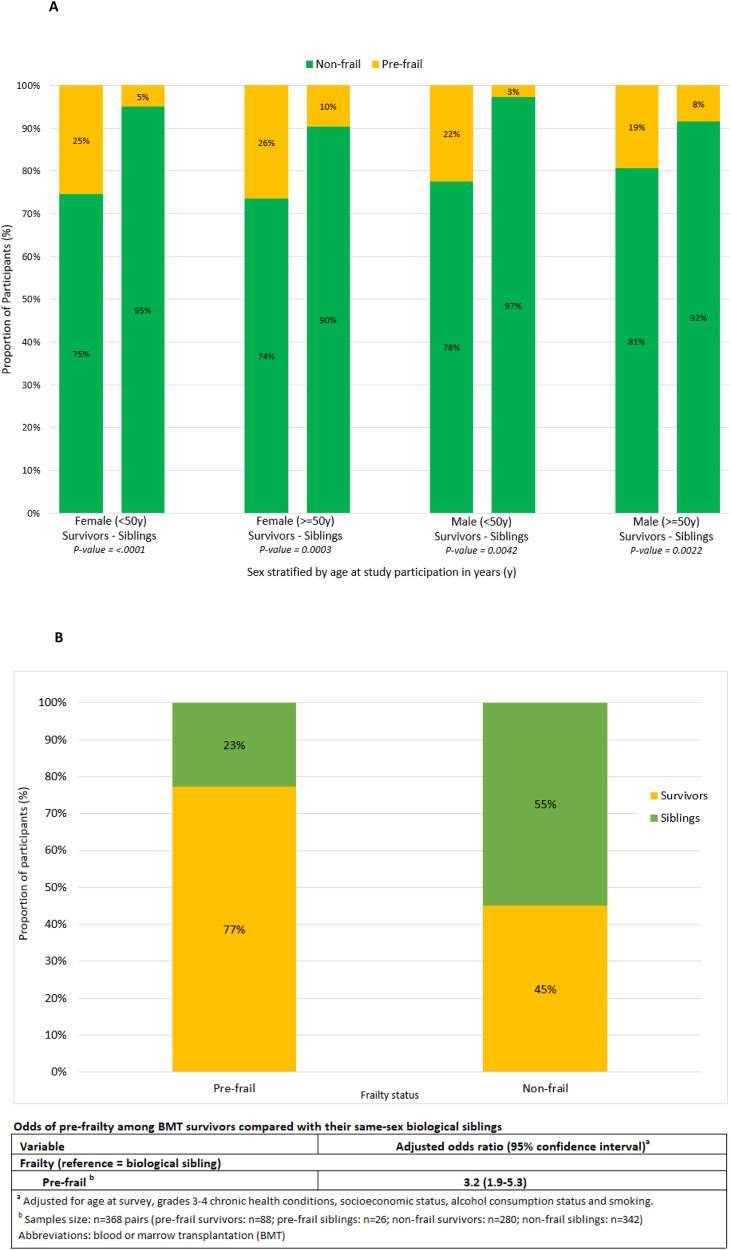
Prevalence and odds of pre-frailty among 763 survivors and paired biologic siblings. **A** Prevalence of pre-frailty by age at survey participation stratified by sex among survivors paired with closest-age and same-sex biologic siblings. **B** Prevalence and odds of pre-frailty among survivors and their paired same-sex biological siblings.

We were able to pair 368 survivors 1:1 with their same-sex biological siblings. The biological siblings were older at survey (mean age: 58.1 years *vs* 49.7 years, *P* < 0.0001), more likely to report smoking (33.2% *vs*. 31.3%, *P* = 0.02) and alcohol consumption (58.1% *vs*. 53.3%, *P* = 0.01), less likely to have lower SES (*P* = 0.04), and less likely to report grade 3–4 chronic health conditions (34.8% *vs*. 63.0%, *P* < 0.0001) (Supplementary Table 6). Pre-frailty was more prevalent among BMT survivors across all ages and both sexes when compared with their siblings (Fig. 1A), as was the prevalence of individual frailty indices, except slowness (Supplementary Table 6). After adjusting for age at survey grades 3–4 chronic health conditions, SES, and alcohol consumption and smoking status, survivors had a 3.2-fold higher odds of being pre-frail (95% CI: 1.9–5.3) compared to their siblings (Fig. 1B).

### Factors associated with pre-frailty after BMT

**Fig. 2:**
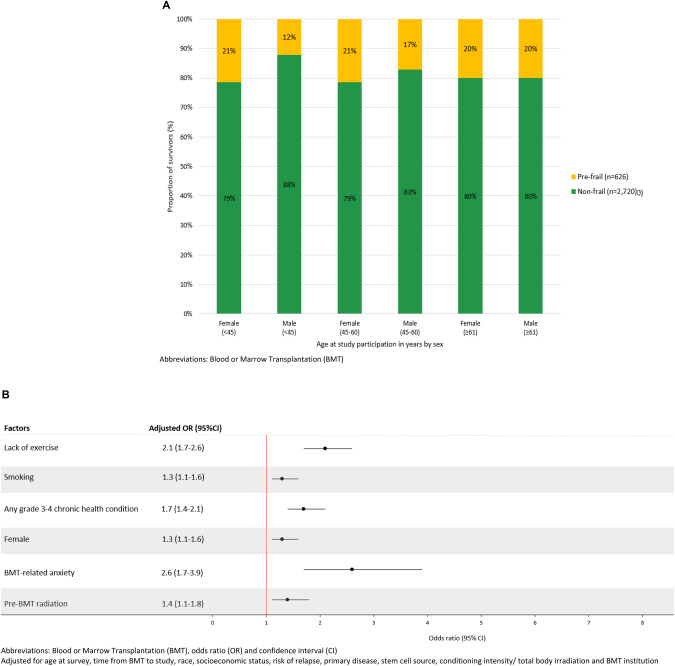
Prevalence and predictors of pre-frailty among 3346 BMT survivors. **A** Prevalence of pre-frailty by age at survey participation stratified by sex among all BMT survivors. **B** Factors associated with pre-frailty after blood or marrow transplantation.

The demographic and clinical characteristics of BMT survivors by pre-frailty status are shown in Table 1. As shown in Fig. 2A, among <45 years-old survivors, females had a higher prevalence of pre-frailty (21% *vs*. 12%) as compared to males. The prevalence of pre-frailty among older survivors (≥61 years-old) was the same for females and males (20%). Factors significantly associated with being pre-frail included lack of exercise (aOR = 2.1, 95% CI: 1.7–2.6; reference: physically active), smoking (aOR = 1.3, 95% CI = 1.1–1.6; reference: never smoker), grade 3–4 chronic health conditions (aOR = 1.7, 95% CI = 1.4–2.1; reference: grades 0–2), female sex (aOR = 1.3, 95% CI = 1.1–1.6), BMT-related anxiety (aOR = 2.6, 95% CI: 1.7–3.9; reference: absent) and pre-BMT radiation (aOR = 1.4, 95% CI = 1.1–1.8) (Fig. 2B).

### Pre-frailty and subsequent late mortality

**Fig. 3:**
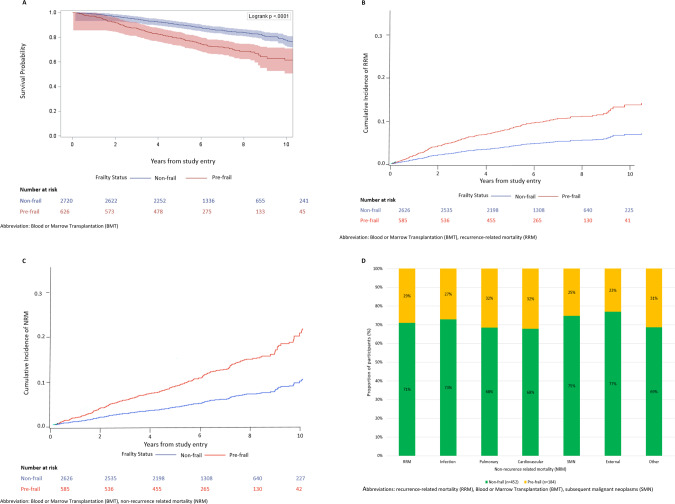
All-cause and cause-specific mortality by pre-frailty status among BMT survivors. **A** All-cause mortality among BMT survivors by pre-frailty status. **B** Recurrence-related mortality among BMT survivors by pre-frailty status. **C** Non-recurrence related mortality among BMT survivors by pre-frailty status. **D** Prevalence of cause-specific mortality by pre-frailty status.

Over a median period of 5 years from BMTSS survey completion, 636 (19.0%) survivors had died (Supplementary Table 7). The 5 years overall survival rate was lower among pre-frail (78.6%) and non-frail (90.2%) survivors, *P* < 0.0001 (Fig. 3A). Pre-frailty was associated with an increased hazard of subsequent all-cause mortality (aHR = 1.6, 95% CI: 1.4–2.0; reference: non-frail) (Table 2). Further, each frailty indicator was associated with a higher risk of all-cause mortality (Supplementary Table 8). No interactions were identified between pre-frailty and the following variables: age at BMT (<45 years *vs*. ≥45 years), primary diagnosis, BMT type, age at survey (<65 years *vs*. ≥65 years), chronic health conditions or stem cell source (data not shown; all *P* > 0.1).

**Table 2:** Hazard of subsequent all-cause and cause-specific late mortality after BMT by pre-frailty status.

	Unadjusted HR (95% CI)			Adjusted a HR (95% CI)		
	All-cause mortality	Non-recurrence-related mortality	Recurrence-related mortality	All-cause mortality	Non-recurrence-related mortality	Recurrence-related mortality
**Pre-frailty status**^**a**^						
Non-frail	Reference	Reference	Reference	Reference	Reference	Reference
Pre-frail	1.9 (1.6–2.3)	1.9 (1.4–2.4)	1.9 (1.3–2.5)	**1.6 (1.4**–**2.0)**	**1.6 (1.2**–**2.1)**	**1.4 (1.0**–**1.9)**

Bold indicates statistically significant differences between groups.

*BMT* blood or marrow transplantation, *cGvHD* Chronic Graft vs Host Disease, *TBI* Total Body irradiation, *HR* hazard ratio, *CI* confidence intervals.

^a^Adjusted for age at completing the survey, time from BMT to completing the survey, sex, race/ethnicity, health insurance, socioeconomic status, primary diagnosis, risk of relapse at BMT, BMT type/cGvHD, stem cell source, condition intensity/TBI, post-BMT relapse, pre-BMT radiation, chronic health conditions, smoking, lack of exercise, alcohol consumption and BMT institution.

The estimated 5 years cumulative incidence of RRM was 4% for the non-frail participants and 9% for pre-frail participants (Fig. 3B). Pre-frail participants had a higher hazard of RRM compared to non-frail participants (aHR = 1.4, 95% CI: 1.0–1.9) (Table 2). The estimated 5 years cumulative incidence of NRM was 3.8% for the non-frail participants and 10.1% for pre-frail participants (Fig. 3C). Pre-frail participants had a higher hazard of NRM compared to non-frail participants (aHR = 1.6, 95% CI: 1.2–2.1) (Table 2). As shown in Fig. 3D, pre-frail patients accounted for 32% of cardiovascular and pulmonary deaths and 23%–31% of other causes of death. Supplementary Table 9 shows the adjusted hazard of cause-specific mortality by frailty status. Of note, pre-frail survivors had a 2.3-fold higher hazard of cardiovascular death (aHR = 2.3, 95% CI = 1.2–4.1; reference: non-frail).

## Discussion

The prevalence of pre-frailty among BMT survivors at a median age of 57 years and a median follow-up of 9 years from BMT was 18.7%. BMT survivors had a 3-fold higher odds of pre-frailty when compared with their same-sex biological siblings. Female sex, pre-BMT radiation, smoking, lack of exercise, BMT-related anxiety, and severe/life-threatening chronic health conditions were associated with pre-frailty. Pre-frail survivors had a 60% higher hazard of all-cause mortality as compared to the non-frail BMT survivors.

We paired survivors between the ages of 18 years and 84 years with their same-sex biological sibling to control for genetic and sociodemographic factors that could affect frailty. No prior study has examined the risk of pre-frailty or frailty among cancer survivors and their biological siblings. A 3.2-fold higher odds of pre-frailty among BMT survivors compared with siblings suggests that the underlying diagnosis and its management place BMT survivors at higher risk for pre-frailty.

Similar to our previous report [1], severe/life-threatening chronic health conditions were associated with greater odds of pre-frailty among the BMT survivors. In addition, in the current study, we found that pre-BMT exposure to radiation increased the odds of pre-frailty by 40%, even after adjusting for grade 3–4 chronic health conditions. This likely reflects subclinical radiation-related organ dysfunction or tissue damage leading to the pre-frail phenotype.

Severe/life-threatening chronic health conditions were associated with a significantly higher risk of pre-frailty. Patients experiencing severe/life-threatening chronic health conditions often contend with heightened disease severity and health challenges. Lack of physical activity was associated with a 2.1-fold increased odds of pre-frailty in our study. While these findings are aligned with previous studies that showed the efficacy of exercise programs in mitigating frailty, causality cannot be determined because of the cross-sectional nature of our study design [12–15]. BMT-related anxiety was associated with 2.6-fold higher odds of pre-frailty, confirming the previous association between pre-frailty and anxiety in the general population and oncology settings [16–19]. Again, causality cannot be determined for the same reasons as described above.

Smoking was associated with 30% higher odds of pre-frailty among BMT survivors in the current report. Many studies have reported mixed results when examining the association between smoking and frailty (there are no reports on pre-frailty) [20, 21]. The biological mechanisms underlying the relationship between smoking and pre-frailty are not fully understood [22]. Smoking depresses muscle protein synthesis and induces an elevated expression of myostatin and muscle atrophy F-box (MAFBx), resulting in muscle catabolism [23]. In addition, smoking is a major risk factor for pulmonary disease [24], which could increase the risk of pre-frailty.

Our results confirm previously reported associations between low SES and pre-frailty in the non-oncology space [2, 25–27]. Low SES likely limits access to healthcare, resulting in worsening of health issues and contributing to pre-frailty without appropriate medical attention [28]. Financial limitations could also prevent individuals from engaging in physical activity and consuming a nutritious diet [29–33], which in turn could result in increased risk of pre-frailty.

Consistent with previous reports in non-cancer populations [34, 35], we found that pre-frailty was associated with higher hazard of mortality and higher hazard of cardiovascular death. To our knowledge, no previous study has examined the association between pre-frailty and subsequent mortality among BMT survivors. In general, pre-frail individuals are at increased risk of hospitalization and falls [14, 36]. Studies among middle-aged and older community-dwelling adults find that pre-frail individuals are more likely to revert back to a robust state than those who are frail [3, 4]. These findings suggest that even among BMT survivors who do not meet frailty criteria, it is important to address the individual frailty components to mitigate adverse outcomes.

Our study needs to be placed in the context of its limitations. Reliance on self-report increases the risk of recall bias. Our criteria for frailty differed slightly from those of Fried et al. [36]; pertinent differences are discussed elsewhere [1]. There is a potential of survival bias, as the time from BMT to survey completion varied between survivors; we addressed this by adjusting for time from BMT to survey completion. Assessment of factors associated with pre-frailty was conducted in a cross-sectional setting, limiting our ability to establish causal relationships. While the association between pre-frailty and subsequent mortality utilized a prospective longitudinal approach, pre-frailty was assessed at a single time point ~9 years post-BMT. The frailty status might have changed during follow-up, leading to non-differential misclassification that is more likely to bias the association between pre-frailty and mortality towards null. These limitations notwithstanding, the current study identifies vulnerable populations at risk of pre-frailty who may benefit from targeted interventions even before the onset of frailty [37, 38].

### Supplementary information

Supplementary materials

## Data Availability

For original data, please contact smitabhatia@uabmc.edu.
