# Supplementary material for: Pre-frailty after blood or marrow transplantation and the risk of subsequent mortality
Source: Leukemia. 2024 Apr 5;38(7):1592–9. doi: 10.1038/s41375-024-02238-2 (PMC11217001; doi:10.1038/s41375-024-02238-2)
Supplement: Supplementary file 1 — Supplementary materials [file 41375_2024_2238_MOESM1_ESM.doc]

**Supplemental materials**

**Pre-frailty after Blood or Marrow Transplantation and the Risk of Subsequent Mortality**

- Supplemental Table 1. Questions from the BMTSS questionnaire
- Supplemental Table 2. Frailty phenotype questions from the BMTSS questionnaire
- Supplemental Table 3. Unadjusted sociodemographic and clinical risk factors for pre-frailty among BMT survivors
- Supplemental Table 4. Unadjusted demographic and clinical risk factors of all-cause late-mortality after BMT
- Supplemental Table 5. Clinical and demographic characteristics of study participants and those who refused participation.
- Supplemental Table 6. Characteristics of BMT survivors and matched biological siblings
- Supplemental Table 7. Clinical and demographic characteristics of study participants by vital status
- Supplemental Table 8. Hazard ratios of all-cause late-mortality among BMT survivors by individual frailty indicator
- Supplemental Table 9. Pre-Frailty and hazard ratio of cause-specific late-mortality
- Supplemental Figure 1. BMTSS survivors’ participation flow diagram

**Supplemental Table 7. Questions from the BMTSS questionnaire**

| **Health behaviors questions** | **Options** | **Comments** |
| --- | --- | --- |
| 1. **Smoking** | | |
| **A.1.** Have you smoked at least 100 cigarettes in your entire life? | - No - Yes | Participants were categorized based on their answer to question **A.1**:   - **Never-smoker:** - No - **Ever-smoker:** - Yes |
| **B. Alcohol Consumption** | | |
| **B.1**. Did you ever drink any alcoholic beverages, such as beer, wine, or hard liquor, on a regular basis, that is, once a week or more for 6 months or longer? | - No - Yes | Participants were categorized based on their answer to question **B.1**:   - - **Ever drinker:**     - Yes     - No |
| **B.2.** During the last 12 months, how often did you have 5 or more (males) or 4 or more (females) drinks containing any kind of alcohol in a single day. | - Never in the past year - 1 or 2 times in the past year - 3 to 11 times in the past year - Once a month - 2 to 3 times a month - Once a week - Twice a week - 3 to 4 times a week - 5 to 6 times a week - Every day. | Participants were categorized based on their answer to questions **B.1** and **B.2**:  If they reported never drink **(B.1)**, they were categorized as   - **Non-drinker**   If they reported ever drink **(B.1)** then   - **Heavy drinker:**    - Females: reported to have ≥4 alcoholic drinks in a single day ≥2 twice a week - Males: reported to have ≥5 alcoholic drinks in a single day at least 3-4 times per week - **Non-heavy drinker**   - Females: reported to have <4 alcoholic drinks in a single day < twice a week - Males: reported to have <5 alcoholic drinks in a single day less than 3-4 times a week |
| **C. Exercise** | | |
| **C.1.** During the past month, did you participate in any physical activities or exercises such as running, calisthenics, golf, gardening, bicycling, swimming, or walking for exercise? | - No - Yes | Participants were categorized based on their answer to question **C.1**:   - **Lack of exercise**   - - Yes     - No |
| **D. BMT-related anxieties** | | |
| **D.1.** Do you currently have anxieties/fears as a result of your BMT? | - No anxiety/fears - Small amount of anxiety/fears - Medium amount of anxiety/fears - A lot of anxiety/fears - Very many, extreme anxiety/fears | Participants were categorized based on their answer to question **D.1:**   - **Absent**   - No anxiety/fears   - Small amount of anxiety/fears - **Present**    - Medium amount of anxiety/fears   - A lot of anxiety/fears   - Very many, extreme anxiety/fears |

Supplemental Table 8. Frailty phenotype questions from the BMTSS questionnaire

| **Indices** | **BMTSS Questionnaire’s Questions/Options** | **Frailty Code** |
| --- | --- | --- |
| **Clinically underweight** | BMI calculated from self-reported weight and height | BMI <18.5 = frail |
| **Exhaustion** | - Questions:   - How much of the time during the past 4 weeks did you feel worn out?   - How much of the time during the past 4 weeks did you feel tired? - Options:   - None of the time   - Some of the time   - A good bit of the time   - All of the time | Participants who answered “a good bit of the time” or “all of the time” to either question were categorized as frail |
| **Low energy expenditure** | - Question:   - How much does your health limit you in running, lifting heavy objects, participating in sports? - Options:   - No, not limited at all   - Yes, limited a little   - Yes, limited a lot | Participants who answered “limited a lot” were categorized as frail |
| **Slowness** | - Questions:   - Does your health limit you in climbing 1 flight of stairs?   - Does your health limit you in walking 1 block? - Options:   - Not limited   - Limited a little   - Limited a lot | Participants who answered “Limited a lot” to either question were categorized as frail |
| **Weakness** | - Question:   - How much has feeling weak in parts of your body distressed/ bothered you during the past 7 days including today? - Options:   - Not at all   - A little bit   - Moderately/Quite a bit   - Extremely | Participants who answered “Extremely” to this question were categorized as frail |

Supplemental Table 9. Unadjusted sociodemographic and clinical risk factors for pre-frailty among BMT survivors

| **Variables of interest** | **Pre-frail** | |
| --- | --- | --- |
|  | **Unadjusted OR (95%CI)** | **P** |
| **Age at completing the survey in years (continuous)** | | |
| Per year increase in age | 1.0 (1.00-1.01) | **0.0229** |
| **BMT era (reference:** **1974-1989)** | | |
| 1990-2004 | 1.1 (0.8-1.5) | 0.7976 |
| 2005-2014 | 1.1 (0.8-1.6) |  |
| **BMT institution (ref: UMN)** | | |
| COH | 1.3 (1.1-1.5) | **<0.0447** |
| UAB | 1.4 (0.9-1.9) |  |
| **Sex (reference: male)** | | |
| Female | 1.3 (1.1-1.5) | **0.0042** |
| **Race/Ethnicity (reference**: **Non-Hispanic White)** | | |
| African American | 0.7 (0.4-1.1) | 0.2052 |
| Hispanic | 0.7 (0.6-1.0) |  |
| Asian | 1.0 (0.7-1.5) |  |
| Other^a^ | 0.9 (0.4-1.5) |  |
| **Health insurance (insured)** | | |
| Not insured | 0.8 (0.5-1.3) | 0.3495 |
| **Socioeconomic status (reference: ≥college and ≥$50,000)** | | |
| <College and <$50,000 | 1.3 (0.9-1.7) | **0.0146** |
| <College and ≥$50,000 | 1.2 (0.8-1.7) |  |
| ≥College and <$50,000 | 1.4 (1.1-1.7) |  |
| **Follow-up from BMT to completing the survey in years (continuous)** | | |
| Per year increase in age | 0.98 (0.97-1.0) | **0.0512** |
| **Primary diagnosis (reference: ALL)** | | |
| AML/MDS | 1.0 (0.7-1.4) | 0.0930 |
| CML | 0.9 (0.6-1.3) |  |
| HL | 0.8 (0.5-1.3) |  |
| NHL | 0.8 (0.6-1.2) |  |
| PCD | 1.2 (0.8-2.1) |  |
| Other^b^ | 0.7 (0.5-1.2) |  |
| **Risk of relapse at first BMT (reference:** **standard risk)** | | |
| High risk | 1.2 (1.0-1.5) | **0.0043** |
| **Post-BMT relapse (reference: no)** | | |
| Yes | 1.2 (0.9-1.7) | 0.1727 |
| **BMT type/ cGvHD (reference: autologous)** | | |
| Allogeneic with cGvHD | 0.9 (0.8-1.1) | 0.2208 |
| Allogeneic without cGvHD | 0.8 (0.6-1.0) |  |
| Allogeneic missing cGvHD | 0.9 (0.4-1.9) |  |
| **Stem cell source (reference: bone marrow/cord blood)** | | |
| Peripheral Stem Cells | 1.3 (1.1-1.6) | **0.0060** |
| **Conditioning intensity/ Total Body Irradiation (reference: NMA/ no TBI)** | | |
| MAC/ no TBI | 0.9 (0.7-1.2) | 0.7923 |
| MAC/ TBI | 1.0 (0.8-1.3) |  |
| NMA/ TBI | 0.9 (0.6-1.3) |  |
| **Pre-BMT radiation (reference: no)** | | |
| Yes | 1.4 (1.1-1.7) | **0.0085** |
| **Chronic health conditions (reference: grades 0 or2)** | | |
| Grades 3 or 4 | 1.8 (1.5-2.1) | **<.0001** |
| **BMT-related anxiety (reference: absent)** | | |
| Present | 2.7 (1.8-3.9) | **<.0001** |
| **Smoking status (reference: never smoker)** | | |
| Ever smoker | 1.4 (1.1-1.6) | **0.0008** |
| **Alcohol consumption status (reference: non-drinker)** | | |
| Non-heavy drinker | 0.9 (0.7-1.0) | 0.1954 |
| Heavy drinker | 0.8 (0.5-1.2) |  |
| **Lack of exercise (reference: no)** | | |
| Yes | 2.1 (1.7-2.7) | **<.0001** |
| ^a^ Race “other” includes Multiracial, American Indian, Pacific Island  ^b^ Primary diagnosis “other” includes severe aplastic anemia (SAA), other Leukemia  Abbreviations: Blood or Marrow Transplantation (BMT), Acute Myeloid Leukemia (AML), Myelodysplastic Syndrome (MDS), Acute lymphoblastic leukemia (ALL), Hodgkin's Lymphoma (HL), plasma cell dyscrasias (PCD), Non-Hodgkin's lymphoma (NHL), Chronic Graft *vs* Host Disease (cGvHD), Myeloablative (MAC), Non-Myeloablative (NMA), chronic myeloid leukemia (CML), Total Body irradiation (TBI), University of Alabama at Birmingham (UAB), City of Hope (COH) and University of Minnesota (UMN), confidence intervals (CI) and odds ratios (OR) | | |

Supplemental Table 10. Unadjusted demographic and clinical risk factors of all-cause late-mortality after BMT

| **Variables of interest** | **Unadjusted HR (95%CI)** | **P** |
| --- | --- | --- |
| **Age at completing the survey in years (continuous)** | | |
| Per year increase in age | 1.04 (1.03-1.05) | **<.0001** |
| **BMT era (reference: 1974-1989)** | | |
| 1990-2004 | 1.0 (0.8-1.3) | 0.1004 |
| 2005-2014 | 1.2 (0.9-1.5) |
| **BMT institution (reference: UMN)** | | |
| COH | 1.2 (1.1-1.4) | **0.0284** |
| UAB | 1.2 (1.9-1.7) |
| **Sex (reference: female)** | | |
| Male | 1.5 (1.3-1.7) | **<.0001** |
| **Race/Ethnicity (reference: Non-Hispanic White)** | | |
| African American | 0.8 (0.6-1.2) | **0.0016** |
| Hispanic | 0.8 (0.6-0.9) |
| Asian | 0.6 (0.4-0.8) |
| Other^a^ | 1.3 (0.9-1.9) |
| **Health insurance (reference: insured)** | | |
| Not insured | 0.7 (0.5-1.0) | **0.0498** |
| **Socioeconomic status (reference: ≥college and ≥$50,000)** | | |
| <College and <$50,000 | 1.4 (1.1-1.6) | **0.0069** |
| <College and ≥$50,000 | 1.0 (0.7-1.3) |
| ≥College and <$50,000 | 1.3 (1.1-1.5) |
| **Follow-up from BMT to completing the survey in years (continuous)** | | |
| Per year increase in follow-up | 0.96 (0.95-0.98) | **<.0001** |
| **Primary diagnosis (reference: ALL)** | | |
| AML/MDS | 1.2 (0.9-1.5) | **<.0001** |
| CML | 1.3 (0.9-1.7) |
| HL | 1.3 (0.9-1.8) |
| NHL | 1.4 (1.1-2.0) |
| PCD | 2.7 (2.0-3.7) |
| Other ^b^ | 0.5 (0.3-0.7) |
| **Risk of relapse at BMT (reference: standard risk)** | | |
| High risk | 1.7 (1.4-1.9) | **<.0001** |
| **BMT type/cGvHD (reference: autologous)** | | |
| Allogeneic with cGvHD | 0.8 (0.7-0.9) | **<.0001** |
| Allogeneic without cGvHD | 0.4 (0.3-0.5) |
| Allogeneic missing cGvHD | 0.5 (0.2-1.3) |
| **Stem cell source (reference: bone marrow/cord blood)** | | |
| Peripheral stem cells | 1.7 (1.5-2.0) | **<.0001** |
| **Conditioning intensity/ total body irradiation (reference:** **NMA/ no TBI)** | | |
| MAC/ no TBI | 1.4 (1.1-1.8) | **0.0184** |
| MAC/ TBI | 1.1 (0.9-1.4) |
| NMA/ TBI | 1.1 (0.7-1.6) |
| **Post-BMT relapse (reference: no)** | | |
| Yes | 2.7 (2.2-3.2) | **<.0001** |
| **Pre-BMT radiation (reference: no)** | | |
| Yes | 1.4 (1.2-1.6) | **0.0002** |
| **Chronic health conditions (reference: grades 0-2)** | | |
| Grades 3-4 | 2.1 (1.8-2.5) | **<.0001** |
| **BMT-related anxiety (reference: absent)** | | |
| Present | 1.2 (0.9-1.6) | 0.3458 |
| **Smoking status (reference: never smoker)** | | |
| Ever smoker | 1.6 (1.3-1.8) | **<.0001** |
| **Alcohol consumption status (reference: non-drinker)** | | |
| Non-heavy drinker | 1.2 (1.1-1.5) | **0.0089** |
| Heavy drinker | 1.2 (0.9-1.6) |
| **Lack of exercise (reference: no)** | | |
| Yes | 1.7 (1.4-2.0) | **<.0001** |
| **Frailty (reference: non-frail)** | | |
| Pre-frail | 1.9 (1.6-2.3) | **<.0001** |
| Abbreviations: Blood or Marrow Transplantation (BMT), Acute Myeloid Leukemia (AML), Myelodysplastic Syndrome (MDS), Acute Lymphoblastic Leukemia (ALL), Hodgkin Lymphoma (HL), non-Hodgkin Lymphoma (NHL), Chronic Myeloid Leukemia (CML), Plasma Cell Dyscrasias (PCD), chronic Graft *vs* Host Disease (cGvHD), Myeloablative (MAC), Non-Myeloablative (NMA), Total Body irradiation (TBI), hazard ratio (HR), confidence intervals (CI), University of Alabama at Birmingham (UAB), City of Hope (COH) and University of Minnesota (UMN) | | |

**Supplemental Table 11. Clinical and demographic characteristics of study participants and those who refused participation**

| **Variables** | **Refusals**  **(n=1,512)** | **Participants**  **(n=3,346)** | **P-Value** |
| --- | --- | --- | --- |
| **Age at BMT in years** | | | |
| Mean (SD) | 37.9 (19.0) | 41.7 (17.1) | **<.0001** |
| **BMT institution, n (%)** | | | |
| COH | 883 (58.4) | 2,057 (61.5) | **<.0001** |
| UMN | 462 (30.6) | 1,051 (31.4) |
| UAB | 167 (11.0) | 238 (7.1) |
| **Sex, n (%)** | | | |
| Female | 627 (41.5) | 1,485 (44.4) | 0.0579 |
| Male | 885 (58.5) | 1,861 (55.6) |
| **Race/Ethnicity, n (%)** | | | |
| African American | 156 (10.3) | 154 (4.6) | **<.0001** |
| Non-Hispanic White | 964 (63.8) | 2,522 (75.4) |
| Hispanic | 230 (15.2) | 401 (12.0) |
| Asian | 144 (9.5) | 178 (5.3) |
| Other ^a^ | 15 (1.0) | 88 (2.6) |
| Missing | 3 (0.2) | 3 (0.1) |
| **Diagnosis, n (%)** | | | |
| ALL | 126 (8.3) | 279 (8.3) | **<.0001** |
| AML/MDS | 293 (19.4) | 785 (23.5) |
| CML | 112 (7.4) | 335 (10.0) |
| HL | 169 (11.2) | 268 (8.0) |
| NHL | 316 (20.9) | 830 (24.8) |
| PCD | 279 (18.4) | 573 (17.2) |
| Other^b^ | 216 (14.3) | 276 (8.3) |
| Missing | 1 (0.1) | 0 (0.0) |
| **BMT Type/ cGvHD, n (%)** | | | |
| Autologous | 793 (52.4) | 1,704 (50.9) | 0.3262 |
| Allogeneic | 719 (47.6) | 1,642 (49.1) |
| **Conditioning intensity/ Total Body Irradiation, n (%)** | | | |
| MAC/ no TBI | 463 (30.6) | 1,048 (31.3) | **<.0001** |
| MAC/ TBI | 401 (26.5) | 1,283 (38.3) |
| NMA/ no TBI | 221 (14.6) | 435 (13.0) |
| NMA/ TBI | 102 (6.7) | 217 (6.5) |
| Missing | 325 (21.5) | 363 (10.9) |
| **Disease status at first BMT, n (%)** | | | |
| High risk | 688 (45.5) | 1,466 (43.8) | **<.0001** |
| Standard risk | 529 (35.0) | 1,487 (44.4) |
| Missing | 295 (19.5) | 393 (11.7) |
| ^a^ Other race included: Multi-racial, Pacific islander, and American Indian  Abbreviation: BMT = blood marrow transplant. TBI = Total Body Irradiation, City of Hope (COH) and University of Minnesota (UMN), Acute lymphoblastic leukemia (ALL), Acute Myeloid Leukemia (AML), Myelodysplastic Syndrome (MDS), chronic myeloid leukemia (CML), Hodgkin's Lymphoma (HL), Non-Hodgkin's lymphoma (NHL), plasma cell dyscrasias (PCD), Myeloablative (MAC), Non-Myeloablative (NMA) and Chronic Graft vs Host Disease (cGvHD)  Bold indicates statistically significant differences between groups | | | |

**Supplemental Table 12. Characteristics of BMT survivors and matched biological siblings**

| **Variables** | **Siblings**  **N = 368** | **BMT survivors**  **N=368** | **P-value** |
| --- | --- | --- | --- |
| **Age at completing the survey in years** | | | |
| Mean (SD) | 58.1 (14.9) | 49.7 (1.6) | **<.0001** |
| **Sex, n (%)** | | | |
| Female | 238 (64.7) | 238 (64.7) | Paired |
| Male | 130 (35.3) | 130 (35.3) |
| **Health insurance, n (%)** | | | |
| Not insured | 5 (1.4) | 4 (1.1) | 0.7373 |
| Insured | 363 (98.6) | 364 (98.9) |
| **Socioeconomic status, n (%)** | | | |
| <College and <$50,000 | 49 (13.3) | 33 (9.0) | **0.0403** |
| <College and ≥$50,000 | 23 (6.3) | 16 (4.3) |
| ≥College and <$50,000 | 91 (24.7) | 120 (32.6) |
| ≥College and ≥$50,000 | 171 (46.5) | 172 (46.7) |
| Missing | 34 (9.2) | 27 (7.3) |
| **Frailty indicators, n (%)** | | | |
| Clinically underweight | 6 (1.6) | 16 (4.3) | **0.0304** |
| Exhaustion | 80 (21.7) | 120 (32.6) | **0.0059** |
| Low energy expenditure | 56 (15.2) | 131 (35.6) | **<.0001** |
| Slowness | 4 (1.1) | 2 (0.5) | 0.4123 |
| Weakness | 4 (1.1) | 13 (3.5) | **0.0392** |
| **Frailty phenotype, n (%)** | | | |
| Non-frail | 342 (92.9) | 280 (76.1) | **<.0001** |
| Pre-frail | 26 (7.1) | 88 (23.9) |
| **Smoking status, n (%)** | | | |
| Never smoker | 244 (66.3) | 253 (68.7) | **0.0216** |
| Ever smoker | 122 (33.2) | 115 (31.3) |
| Missing | 2 (0.5) | 0 (0.0) |
| **Alcohol consumption status, n (%)** | | | |
| Non-drinker | 153 (41.6) | 171 (46.5) | **0.0160** |
| Non-heavy drinker | 190 (51.6) | 188 (51.1) |
| Heavy drinker | 23 (6.3) | 8 (2.2) |
| Missing | 2 (0.5) | 1 (0.2) |
| **Lack of exercise, n (%)** | | | |
| No | 282 (76.6) | 319 (86.7) | 0.0996 |
| Yes | 61 (16.6) | 49 (13.3) |
| Missing | 25 (6.8) | 0 (0.0) |
| **Chronic health conditions, n (%)** | | | |
| Grades 3 or 4 | 128 (34.8) | 232 (63.0) | **<.0001** |
| Abbreviations: blood or marrow transplantation (BMT) | | | |

Supplemental Table 7. Clinical and demographic characteristics of study participants by vital status

| **Variables of interest** | **Vital Status** | | **P-value** |
| --- | --- | --- | --- |
|  | **Alive**  **N= 2,710 (81.0%)** | **Deceased**  **N=636 (19.0%)** |  |
| **Age at completing the survey in years** | | | |
| Median (IQR) | 56 (42-64) | 60 (50-67) | <.0001 |
| **Age at completing the survey in years** | | | |
| <65 | 2058 (75.9) | 409 (64.3) | <.0001 |
| ≥65 | 652 (24.1) | 227 (35.7) |  |
| **Follow-up from completing the survey to death or end of follow-up in years** | | | |
| Median (IQR) | 6 (4-7) | 4 (2-7) | <.0001 |
| **BMT era, n (%)** | | | |
| 1974-1989 | 196 (7.2) | 87 (13.7) | <.0001 |
| 1990-2004 | 1,135 (41.9) | 312 (49.1) |  |
| 2005-2014 | 1,379 (50.9) | 237 (37.3) |  |
| **Institution, n (%)** | | | |
| COH | 1,637 (60.4) | 420 (66.0) | 0.0062 |
| UMN | 865 (31.9) | 186 (29.3) |  |
| UAB | 208 (7.7) | 30 (4.7) |  |
| **Sex, n (%)** | | | |
| Female | 1,255 (46.3) | 230 (36.2) | <.0001 |
| Male | 1,455 (53.7) | 406 (63.8) |  |
| **Race/Ethnicity, n (%)** | | | |
| African American | 131 (4.8) | 23 (3.6) | 0.0268 |
| Asian | 156 (5.8) | 22 (3.5) |  |
| Hispanic | 335 (12.4) | 66 (10.4) |  |
| Non-Hispanic White | 2,019 (74.5) | 503 (79.1) |  |
| Other^a^ | 66 (2.4) | 22 (3.5) |  |
| Missing | 3 (0.1) | 0 (0.0) |  |
| **Health insurance, n (%)** | | | |
| Not insured | 94 (3.5) | 29 (4.6) | 0.1823 |
| Insured | 2,613 (96.4) | 604 (95.0) |  |
| Missing | 3 (0.1) | 3 (0.4) |  |
| **Socioeconomic status, n (%)** | | | |
| <College and <$50,000 | 379 (14.0) | 118 (18.6) | 0.0014 |
| <College and ≥$50,000 | 186 (6.9) | 32 (5.0) |  |
| ≥College and <$50,000 | 788 (29.1) | 213 (33.5) |  |
| ≥College and ≥$50,000 | 1,095 (40.4) | 227 (35.7) |  |
| Missing education and/or income | 262 (9.7) | 46 (7.2) |  |
| **Age at BMT in years** | | | |
| Median (IQR) | 43 (26-55) | 50 (38-59) | <.0001 |
| **Follow-up from BMT to completing the survey in years** | | | |
| Median (IQR) | 9 (6-16) | 7 (5-13) | <.0001 |
| **Primary diagnosis, n (%)** | | | |
| ALL | 232 (8.6) | 47 (7.4) | <.0001 |
| AML/MDS | 662 (24.4) | 123 (19.3) |  |
| CML | 252 (9.3) | 83 (13.0) |  |
| HL | 221 (8.1) | 47 (7.4) |  |
| NHL | 665 (24.5) | 165 (25.9) |  |
| PCD | 417 (15.4) | 156 (24.5) |  |
| Other^b^ | 261 (9.6) | 15 (2.4) |  |
| **Risk of relapse at first BMT, n (%)** | | | |
| High risk | 1,110 (41.0) | 356 (56.0) | <.0001 |
| Standard risk | 1,240 (45.8) | 247 (38.8) |  |
| Missing | 360 (13.2) | 33 (5.2) |  |
| **Post-BMT relapse, n (%)** | | | |
| No | 2,532 (93.4) | 469 (73.7) | <.0001 |
| Yes | 170 (6.3) | 93 (14.6) |  |
| Missing | 8 (0.3) | 74 (11.6) |  |
| **BMT type/ cGvHD, n (%)** | | | |
| Autologous | 1,307 (48.2) | 397 (62.4) | <.0001 |
| Allogeneic with cGvHD | 695 (25.6) | 157 (24.7) |  |
| Allogeneic without cGvHD | 664 (24.5) | 80 (12.6) |  |
| Allogeneic missing cGvHD | 44 (1.6) | 2 (0.3) |  |
| **Stem cell source, n (%)** | | | |
| Bone Marrow/Cord Blood | 940 (34.6) | 195 (30.7) | 0.0640 |
| Peripheral Stem Cells | 1,769 (65.3) | 441 (69.3) |  |
| Missing | 1 (0.1) | 0 (0.0) |  |
| **Conditioning intensity/ Total Body Irradiation, n (%)** | | | |
| MAC /No TBI | 864 (31.9) | 184 (28.9) | <.0001 |
| MAC/TBI | 1,000 (36.9) | 283 (44.5) |  |
| NMA/No TBI | 386 (14.2) | 49 (7.7) |  |
| NMA/TBI | 192 (7.1) | 25 (3.9) |  |
| Missing | 268 (9.9) | 95 (14.9) |  |
| **Pre-BMT radiation, n (%)** | | | |
| Yes | 349 (12.9) | 121 (19.0) | 0.0001 |
| No | 2,124 (78.4) | 473 (74.4) |  |
| Missing | 237 (8.7) | 42 (6.6) |  |
| **Chronic health conditions, n (%)** | | | |
| Grades 3 or 4 | 1,477 (54.5) | 409 (64.3) | <.0001 |
| **Frailty indicators, n (%)** | | | |
| Clinically underweight | 69 (2.6) | 16 (2.5) | 0.9637 |
| Exhaustion | 762 (28.1) | 171 (26.9) | 0.5799 |
| Low energy expenditure | 751 (27.7) | 327 (51.4) | <.0001 |
| Slowness | 85 (3.1) | 76 (11.9) | <.0001 |
| Weakness | 125 (4.6) | 33 (5.2) | 0.5254 |
| **Frailty phenotype, n (%)** | | | |
| Non-frail | 2,268 (83.7) | 452 (71.1) | <.0001 |
| Pre-frail | 442 (16.3) | 184 (28.9) |  |
| **BMT-related anxiety, n (%)** | | | |
| Present | 96 (3.5) | 18 (2.8) | 0.3932 |
| Absent | 2,588 (95.5) | 606 (95.3) |  |
| Missing | 26 (1.0) | 12 (1.9) |  |
| **Smoking status, n (%)** | | | |
| Never smoker | 1,813 (66.9) | 319 (50.2) | <.0001 |
| Ever smoker | 886 (32.7) | 311 (48.9) |  |
| Missing | 11 (0.4) | 6 (0.9) |  |
| **Alcohol consumption status, n (%)** | | | |
| Non-drinker | 1,242 (45.8) | 191 (30.0) | <.0001 |
| Non-heavy drinker | 1,349 (49.8) | 380 (59.7) |  |
| Heavy drinker | 106 (3.9) | 59 (9.3) |  |
| Missing | 13 (0.5) | 6 (0.9) |  |
| **Lack of exercise, n (%)** | | | |
| No | 2,292 (84.6) | 533 (83.8) | 0.0446 |
| Yes | 406 (15.0) | 102 (16.0) |  |
| Missing | 12 (0.4) | 1 (0.2) |  |
| **Cause of death, n (%)** | | | |
| Non-recurrence | - | 305 (48.0) | - |
| Recurrence | - | 183 (28.8) |  |
| Unknown | - | 135 (21.2) |  |
| External | - | 13 (2.0) |  |
| ^a^ Race “other” includes Multiracial, American Indian and Pacific Islander  ^b^ Primary diagnosis “other” includes severe aplastic anemia (SAA), Other leukemia, and other  Abbreviations: Blood or Marrow Transplantation (BMT), Acute Myeloid Leukemia (AML), Myelodysplastic Syndrome (MDS), Acute lymphoblastic leukemia (ALL), Hodgkin lymphoma (HL), non-Hodgkin lymphoma (NHL), Chronic Myeloid Leukemia (CML), Plasma Cell Dyscrasias (PCD), chronic Graft *vs* Host Disease (cGvHD), Myeloablative conditioning (MAC), Non-Myeloablative (NMA), Total Body irradiation (TBI), University of Alabama at Birmingham (UAB), City of Hope (COH) and University of Minnesota (UMN), interquartile range (IQR) | | | |

Supplemental Table 8. Hazard ratios of all-cause late-mortality among BMT survivors by individual frailty indicator

| **Frailty indicators** | **Adjusted** ^a^ **HR (95% CI) ^a^** |
| --- | --- |
| **Clinically underweight (reference: no)** | **1.6 (1.0-2.7)** |
| **Exhaustion (reference: no)** | **1.2 (1.0-1.3)** |
| **Low energy expenditure (reference: no)** | **1.8 (1.5-2.1)** |
| **Slowness (reference: no)** | **1.7 (1.3-2.2)** |
| **Weakness (reference: no)** | **1.2 (1.0-1.7)** |
| ^a^ Adjusted for age at completing the survey, time from BMT to completing the survey, sex, race/ethnicity, health insurance, socioeconomic status, primary diagnosis, risk of relapse at BMT, BMT type/cGvHD, stem cell source, condition intensity/TBI, post-BMT relapse, pre-BMT radiation, chronic health conditions, smoking, lack of exercise, BMT institution and alcohol consumption.  Abbreviations: Blood or Marrow Transplantation (BMT), Chronic Graft vs Host Disease (cGvHD), Total Body irradiation (TBI), hazard ratio (HR) and confidence intervals (CI) | |

Supplemental Table 9. Pre-frailty and hazard ratio of cause-specific late-mortality

| **Variable of Interest** | **Adjusted HR (95% CI)** | **Number of Deaths (%)** |
| --- | --- | --- |
| **Pulmonary related mortality** | |  |
| Non-frail | Reference | 13 (68.4) |
| Pre-frail | 1.9 (0.6-5.9) | 6 (31.6) |
| **SMNs related mortality** | |  |
| Non-frail | Reference | 83 (74.8) |
| Pre-frail | 1.3 (0.8-2.1) | 28 (25.2) |
| **Infection related mortality** | |  |
| Non-frail | Reference | 43 (72.9) |
| Pre-frail | 1.6 (0.9-2.9) | 16 (27.1) |
| **Cardiovascular** **related mortality** | |  |
| Non-frail | Reference | 56 (68.3) |
| Pre-frail | 2.3 (1.2-4.1) | 26 (31.7) |
| Adjusted for age at completing the survey, time from BMT to completing the survey, sex, race/ethnicity, health insurance, socioeconomic status, primary diagnosis, risk of relapse at BMT, BMT type/cGvHD, stem cell source, condition intensity/TBI, post-BMT relapse, pre-BMT radiation, chronic health conditions, smoking, lack of exercise, alcohol consumption and BMT institution. The cardiovascular model was also adjusted for cardiovascular risk factors (e.g., diabetes, hypertension and dyslipidemia).  Abbreviations: Blood or Marrow Transplantation (BMT), Chronic Graft vs Host Disease (cGvHD), Total Body irradiation (TBI), hazard ratio (HR) and confidence intervals (CI) | | |

Supplemental Figure 2. BMTSS survivors’ participation flow diagram


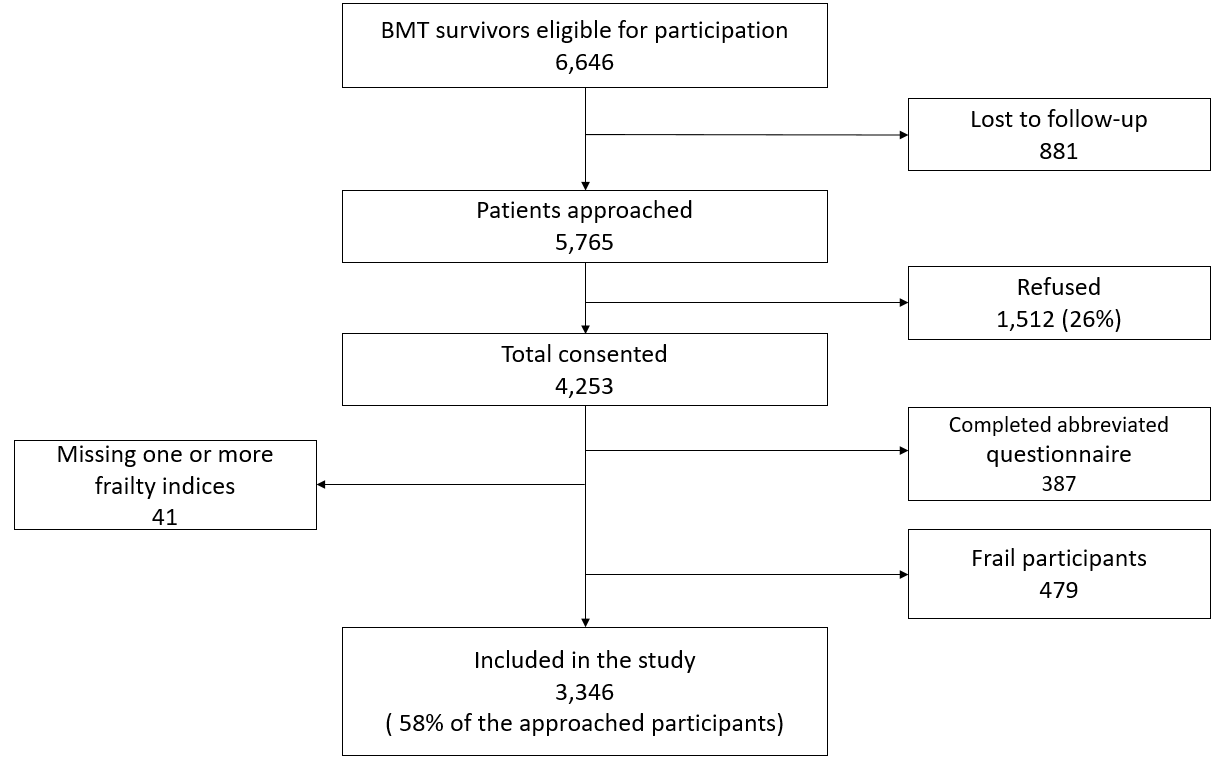


Abbrevitation: Blood or Marrow Transplantation Survivor Study (BMTSS), Blood or Marrow Transplantation (BMT)
